# The effect of probiotic supplementation on episiotomy wound healing among primiparous women: a triple-blind randomized clinical trial

**DOI:** 10.1186/s12906-023-03980-3

**Published:** 2023-05-05

**Authors:** Derakhshan Abdollahpour, Aziz Homayouni-Rad, Roghaiyeh Nourizadeh, Sevil Hakimi, Esmat Mehrabi

**Affiliations:** 1 Student Research Committee, Midwifery Department, Faculty of Nursing and Midwifery, Tabriz University of Medical Sciences, Tabriz, Iran; 2 Department of Food Science and Technology, Faculty of Nutrition & Food Science, Tabriz University of Medical Sciences, Tabriz, Iran; 3 Midwifery Department, Faculty of Nursing and Midwifery, Tabriz University of Medical Sciences, Tabriz, Iran

**Keywords:** Episiotomy, Lactobacillus casei, Probiotics, Wound healing

## Abstract

**Background:**

Probiotics increase the defense power of immune system and accelerate the wound healing process by anti-inflammatory mechanisms at the wound site. The present study aimed at evaluating the effect of Lactobacillus casei oral supplementation on episiotomy wound healing among primiparous women.

**Methods:**

This triple-blind randomized clinical trial was performed on 74 primiparous women delivered in Alzahra Hospital, Tabriz, Iran. Participants with mediolateral episiotomy (incision length equal to and less than 5 cm) were randomly assigned to the probiotic and placebo groups. The probiotic group received Lactobacillus casei 431 with 1.5 * 10^9^ colony-forming unit /capsule once a day from the day after birth to 14 days. Wound healing as a primary outcome was measured by Redness, Edema, Ecchymosis, Discharge, Approximation and pain as a secondary outcome by the Visual Analogue Scale before discharge, 5 ± 1 and 15 ± 1 days after birth. The data were analyzed using independent t-test and repeated measures one way analysis of variance.

**Results:**

The mean (standard deviation: SD) score of wound healing in the probiotic group altered from 4.91(1.86) before discharge to 1.55 (0.99) during 5 ± 1 days after birth and reached to 0.95 (0.27) during 15 ± 1 days after birth. Further, the mean (SD) score of wound healing in the placebo group altered from 4.62 (1.99) before discharge to 2.80 (1.20) during 5 ± 1 days after birth and reached to 1.45(0.71) during 15 ± 1 days after birth (adjusted mean difference: -0.50, confidence interval 95%: -0.96 to -0.05, P = 0.03).

**Conclusion:**

Lactobacillus casei oral supplementation is effective in healing episiotomy wounds. It is suggested to evaluate the effect of topical use of Lactobacillus casei on episiotomy repair and pain in further studies.

**Trial registration:**

Iranian Registry of Clinical Trials (IRCT): IRCT20170506033834N7. Date of registration: 11/08/2021.

## Introduction

Episiotomy means making surgical incision in the perineal muscles during the second stage of labor for widening the pelvic outlet and increasing the speed of fetal head exit. Episiotomy is used in cases with the possibility of large tears, shoulder dystocia, forceps or vacuum delivery, etc. [1]. The prevalence of episiotomy varies from 8% in the Netherlands to 20% in the United Kingdom and 50% in the United States [2]. The rate of episiotomy is reported to be less than 30% in Western countries and more than 70% in East Asian countries [3]. It is widely performed in Asian countries due to their short perineum and proneness to rupture of the perineum. Also, it is mostly because of birth attendants used to have a lot of interventions during labor and delivery [4]. The prevalence of episiotomy among primiparous women in Iran has been reported to be 97.3% [5].

The slow healing process of an episiotomy wound may increase pain and the likelihood of infection, since the incision is located where the wound is more likely to be infected with vaginal and rectal bacteria.^1^ Women with an episiotomy do not need routine use of antibiotics to prevent infection, because the widespread use of antibiotics can lead to the development of antibiotic resistance [6].

Bifidobacteria and lactobacilli are common probiotics with beneficial effects on skin repair [7]. Probiotics can increase the strength of the immune system and accelerate the wound healing process by anti-inflammatory mechanisms at the wound site [8]. Gut probiotics can influence wound healing through three physiological routes, including the central nervous system, modulating immune system, and the transfer of nutrients through the blood flow. Probiotics can produce neuroactive molecules or regulate the secretory activity of endocrine cells, leading to the release of neuromodulators with the potential to repair the tissue. Gut probiotics can stimulate the polymorphonuclear lymphocytes recruitment to the damaged tissue. At the same time, they are determinant in activating innate and adaptive immune responses through T-cells in the lymph nodes. Further, beneficial intestinal bacteria increase the absorption of nutrients necessary for wound healing. An increase in number of beneficial Lactobacilli compared to the level of pathogenic Coriobacteriales and Clostridiales in gut bacterial population leads to the inhibition of inflammation. Therefore, the systemic administration of certain probiotics strains can combat skin inflammation by altering the composition of the gut microbiome [9–11].

In the literature review, among the probiotics, anti-inflammatory effects and angiogenic properties have been reported for Lactobacillus casei, Lactobacillus reuteri, Lactobacillus acidophilus, and Lactobacillus brevis [12–15]. Most laboratory studies examined the benefits of using topical probiotics in wound healing by reducing bacterial load and increasing wounded tissue repair in rodent [16–20]. However, oral consumption of probiotics affects both local and systemic immune system. Oral use of probiotics improves intestinal microbial flora and absorption of nutrients, including vitamins, minerals, and cofactors required for tissue repair, increases Foxp3 + Treg cells in skin lymph nodes and regulates positive expression of interleukin-10, reduces tissue damage in wounds, and decreases inflammation [21, 22]. In human studies, topical probiotics have been mainly used to repair burn wounds, surgical incisions, and diabetic wounds [23–26]. Given the lack of studies on the systemic impact of lactobacilli on the wound healing process and its resultant pain, and considering that no study has been found to measure the effect of probiotics on episiotomy wound healing, the present study aimed to assess the effect of Lactobacillus casei oral supplementation on episiotomy wound healing as primary outcome and its resultant pain as secondary outcome.

## Method

### Study design and participants

This triple-blind randomized clinical trial was conducted on 74 women delivered in Alzahra Hospital, Tabriz. The inclusion criteria were primiparous women with mediolateral episiotomy, singleton delivery, body mass index less than 30, membrane rupture ≤ 18 h, not habitual use of probiotic products, having no gastrointestinal problems, having no history of asthma and allergies to certain substances, episiotomy site length ≤ 5 cm, non-vegetarian diet, and having no history of diseases impairing wound healing, such as immunodeficiency disorder, connective tissue disorder, diabetes, and anemia. The exclusion criteria were curettage of the uterus due to postpartum hemorrhage, and other indications for antibiotic use in postpartum.

### Sample size

The sample size was calculated based on the wound healing variable in the study of Mohammadi et al. [27], using G-Power software. Considering M_1_ = 1.6, M_2_ = 3.0, SD1 = 1.3, SD2 = 1.6, α = 0.05, and Power = 90%, sample size was obtained 20 in each group. Further, based on the variable of pain and regarding M_1_ = 1.2, M_2_ = 2.6, SD1 = 1.6, SD2 = 2.1, α = 0.05, and Power = 90%, the sample size of 32 was estimated in each group. Therefore, considering more sample size (n = 32) in each group and 15% attrition, the final sample size of 37 was obtained in each group.

### Sampling

After registering the study on the website of the Iranian Registry of Clinical Trials (IRCT) (IRCT20170506033834N7) and receiving a referral letter, the researcher (first author) introduce herself to the hospital officials and attended the delivery ward. After birth, she introduced herself to primiparous women with mediolateral episiotomy with an incision length of 5 cm or less, and after evaluating other inclusion criteria, explained the objectives and method of the study to postpartum women. The eligible women willing to participate in the study completed the written informed consent form. The researcher filled out the demographic and obstetric characteristics based on their medical records. Participants were assigned into the probiotic and placebo groups in a ratio of 1: 1 by block randomization using Random Allocation Software (RAS) with a block size of 4 and 6. Envelopes were prepared according to the number of samples and capsules were placed inside the envelopes. Each envelope was numbered from 1 to 74. The envelopes were opened in the order in which the participants entered the study and the type of intervention was determined. Their preparation was performed based on the allocation sequence by a non-involved person in the sampling. In the present study, the researcher, participants, and outcome assessor were blinded.

### Intervention

The probiotic group received Lactobacillus casei 431 with 1.5* 10^9^ colony-forming unit (CFU)/capsule once a day [28], from the day after birth, for two weeks. Participants received the drug in two stages, as the envelopes contained 5 capsules (consumption of one capsule daily after lunch) for the first 5 days after birth and in the next stage, 9 capsules were distributed among the participants for the next days. The placebo group received maltodextrin, 150 mg/day in the same way. The probiotic capsule and placebo were similar in shape, color, and odor. All probiotic and placebo capsules were provided by Tak Gen Zist Pharmaceutical Company, Tehran, Iran. Both groups received routine inpatient treatments. Participants in both groups were trained on how to care of the perineum and sutures, personal hygiene, and nutrition. Postpartum care was the same for both groups according to the national protocol [29]. A sheet containing a table with the days of the week was given to the individuals to mark it in the appropriate place each day after consumption. A phone number was provided to patients to contact if they have any questions or problems.

How they use the drugs and the side effects were controlled by phone during the treatment period. In addition, 10 acetaminophen tablets (500 mg) and a checklist about the number of painkillers used after birth were provided to both groups and they were asked to bring the completed checklist of supplementation, painkiller, envelopes related to the used supplementation at the visit of 5 ± 1 and 15 ± 1 days after birth. The wound healing rate was assessed using Redness, Edema, Ecchymosis, Discharge, Approximation (REEDA) scale and episiotomy pain rate by Visual Analog Scale (VAS) before discharge and 5 ± 1 and 15 ± 1 days after birth. The participants visited in the hospital by the sixth author (outcome assessor).

### Data Collection Tools

The data were collected using demographic and obstetric profile, REEDA, VAS and side events checklist.

Demographic and obstetric characteristic included variables of age, level of education, employment status, adequacy of household income, length of labor stages, number of stitches, birth assistant, etc.

The REEDA scale consists of the following five domains and each of which is scored from 0 to 3:

#### Redness

0 is considered for no redness, 1 for redness at a distance of 0.25 cm from the wound edge, 2 for redness at a distance of 0.5 cm from the wound edge, and 3 for redness beyond 0.5 cm.

#### Edema

0 is regarded for no edema, 1 for less than 1 cm from the perineal incision area, 2 for 2 cm from the perineal incision area, and 3 for more than 2 cm from the perineal incision area.

#### Ecchymosis

0 for no ecchymosis, 1 for within 0.25 cm bilaterally or 0.5 cm unilaterally, 2 for within 1 cm bilaterally or 2 cm unilaterally, and 3 for more than 2 cm bilaterally or more than 3 cm unilaterally.

#### Discharge

0 for no discharge, 1 for serous, 2 for purulent serous, and 3 for bloody- purulent discharge.

#### Approximation

0 for fully closed wound, 1 for skin separation ≤ 3 mm, 2 for skin and subcutaneous fat separation, 3 for subcutaneous fat and fascia layer separation.

All variables (except discharge) were measured using a disposable syringe 5 cc, and the sum of scores was determined. Low scores indicate better status. In order to determine the reliability of the instrument in examining perineal repair during measurements for four times from 6 h to ten days after birth, kappa correlation coefficient was reported 0.75–0.88 for discharge, 0.46 for edema, 0.42 for ecchymosis, and 0.66 for redness [30].

The pain intensity was measured using VAS, which is a 10 cm straight horizontal line, scaled from 0 to 10, as 0 represents “absolute painlessness” and 10 indicates “unbearable pain”. The distance in this line is interpreted as 0 for no pain, 1–3 for mild pain, 4–6 for moderate pain, 7–9 for severe pain, and 9–10 for very severe pain [31]. Its validity and reliability have already been confirmed [32].

In the current research, the side events were extracted through literature review and were listed in a checklist which included bloating, diarrhea, nausea, and headache. Any reported side events by participants were also recorded.

### Data analysis

The collected data were analyzed using SPSS_24_ software and data normality was assessed using Shapiro-wilk test. Intention-to-treat (ITT) was applied for analyzing results. In this study, wound healing was considered as the primary outcome and pain as the secondary outcome. Independent t-test and repeated measures one way analysis of variance (RMANOVA) were employed to compare episiotomy repair and pain between the two groups.

## Results

**Fig. 1:**
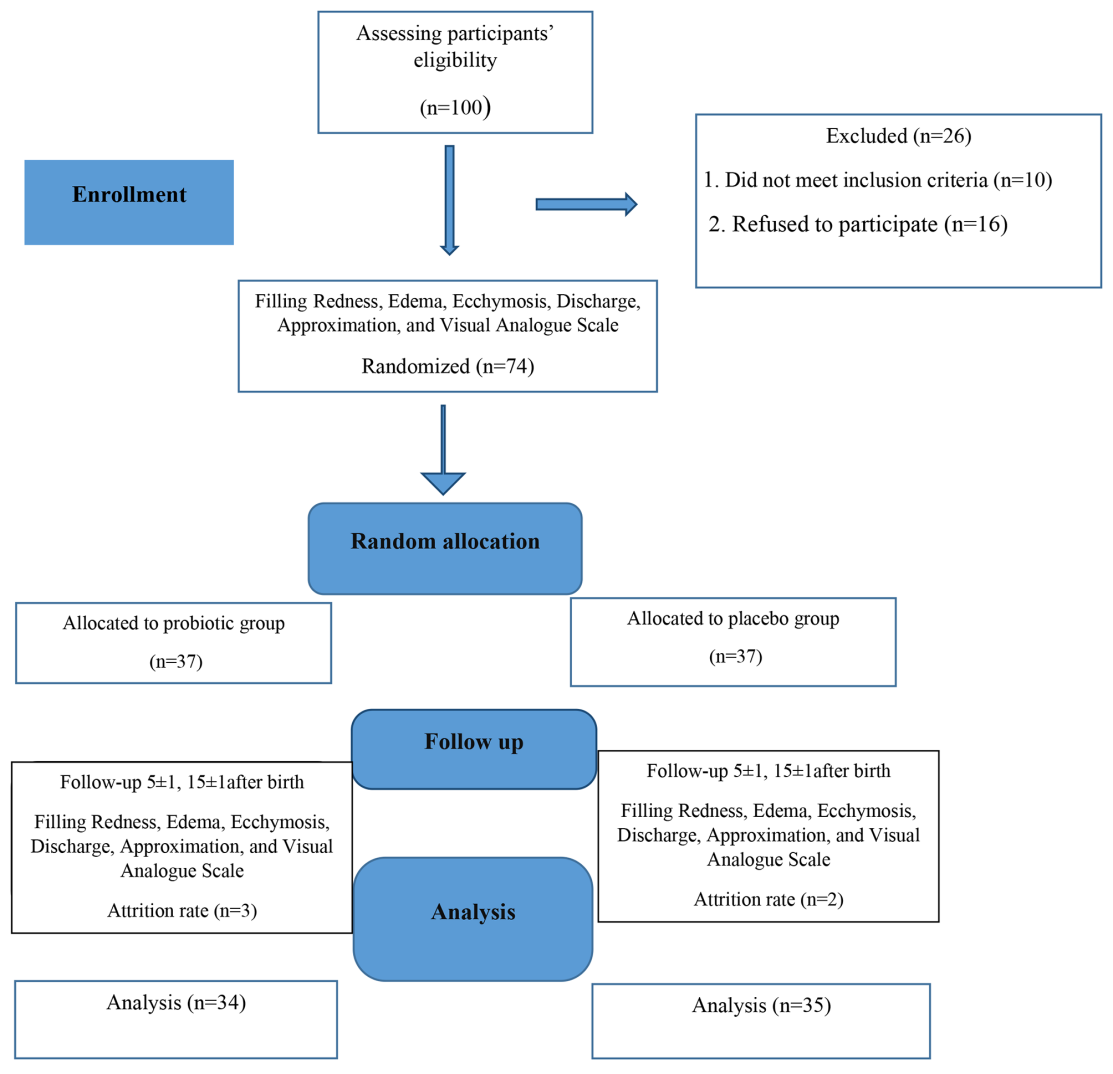
Flowchart of the study

Participants entered the study from July 2021 to January 2022. Of the 100 primiparous women who gave birth, 74 eligible women were randomly assigned to two groups (n = 37). Two women in the probiotic group refused to take drugs due to Covid- 19 infection and did not follow due to quarantine, and one was excluded from the study, due to unwillingness to continue the participation. Two persons in the placebo group were excluded from the study, due to non-referral on follow-up days, and the information of 69 women was finally analyzed (Fig. 1).

The mean (SD) age of women was 25.75 (5.48) in the probiotic group and 27.05(5.57) in the placebo group (P = 0.317). The mean (SD) of body mass index was 25.07 (3.72) in the probiotic group and 25.72 (2.58) in the placebo group (P = 0.387). The majority of participants in the probiotic (91.9%) and placebo (94.6%) groups were housekeeper. More than half of the participants in the probiotic (54.1%) and placebo (64.9%) groups were under diploma. Most of the participants (81.1% in the probiotic group and 78.4% in the placebo group) reported their family income as sufficient. The average number of skin sutures was 4.83 (1.11) in the probiotic group and 4.94 (0.99) in the placebo group (p = 0.662). In general, there was no statistically significant difference between the two groups in terms of demographic and obstetrics characteristics (Table 1).

**Table 1:** The comparison of obstetrics and obstetrics characteristics between probiotic and placebo groups

Variable	Probiotic group (n = 37) N(%)	Placebo group (n = 37) N(%)	P-value
**Age (year)†**	25.75(5.48)	27.05 (5.57)	0.317*
**Body mass index (kg/m** ^**2**^ **)†**	25.07(3.72)	25.72(2.58)	0.387*
**Level of education**			
High school Diploma University	20(54.1) 13(35.1) 4(10.8)	24(64.9) 9(24.3) 4(10.8)	0.580**
**Occupation**			
Housekeeper Employed Worker	34(91.9) 1(2.7) 2(5.4)	35(94.6) 1(2.7) 1(2.7)	0.84**
**Family income level**			
Less than enough Enough More than enough	7(18.9) 30(81.1) 0(0)	5(13.5) 29(78.4) 3(8.1)	**0.187
**Birth assistant** †			
Midwife Obstetrics assistant	13(35.1) 24(64.9)	11(29.7) 26(70.3)	**0.484
**Episiotomy incision length (cm)†**	3.75(0.59)	3.63(0.65)	0.405*
**Number of skin sutures †**	4.83(1.11)	4.94(0.99)	0.662*
**Newborn’s weight (grams)†**	3385(363.02)	3336.48(436.15)	0.604*
**Newborn’s head circumference (cm) †**	33.82(0.63)	34.63(1.17)	0.397*
**Length of the first stage of labor (minute)†**	263.37(145.79)	298.24(160.55)	0.321*
**Length of the second stage of labor (minute)†**	52.70(27.95)	52.32(27.28)	0.953*
**Length of the third stage of labor (minute)†**	12.75(11.02)	11.54(5.11)	0.518*
**Start of activity (day) †**	12.11(7.10)	12.75(5.72)	0.669*

† Mean (standard deviation),* Independent t-test, **Chi-square

The mean (SD) score of wound healing in the probiotic group altered from 4.91(1.86) before discharge to 1.55 (0.99) during 5 ± 1 days after birth and reached to 0.95 (0.27) during 15 ± 1 days after birth. Further, the mean (SD) score of wound healing in the placabo group altered from 4.62(1.99) before discharge to 2.80 (1.20) during 5 ± 1 days after birth and reached to 1.45(0.71) during 15 ± 1 days after birth (adjusted mean difference [AMD]: -0.50, confidence interval [CI] 95%: -0.96 to -0.05, P = 0.03). The episiotomy wound had a healing process in both groups over time, which was significantly faster in the intervention group compared to the control group (Table 2).

**Table 2:** The comparison of mean score of wound healing in probiotic and placebo groups over time

Group Time	Probiotic group (n = 34) Mean (SD)	Placebo group (n = 35) Mean (SD)	Mean difference (95% Confidence interval)	P-value
**Before discharge**	4.91(1.86)	4.62(1.99)	0.29(-0.61 to 1.20)	0.52*
**5 ± 1 days after birth**	1.55(0.99)	2.80(1.20)	-1.24(-1.87 to -0.61)	0.006*
**15 ± 1 days after birth**	0.95(0.27)	1.45(0.71)	-0.50(-0.96 to -0.05)†	0.03**

†Adjusted mean difference after controlling the baseline score, *Independent t –test, **Repeated measures ANOVA

The mean (SD) score of used painkillers was 6.88 (2.73) in the probiotic group and 8.77 (4.04) in the placebo group (P = 0.141). Mean (SD) score of pain before discharge in the probiotic group changed from 4.05 (2.38) to 1.47 (0.64) on 5 ± 1 days after birth and 0.37 (0.17) on 15 ± 1 days after birth. Further, the mean (SD) score of pain before discharge in the placebo group decreased from 3.97 (1.44) to 1.95 (0.35) on 5 ± 1 days after birth and 0.46 (0.22) on 15 ± 1 days after birth, after controlling the effect of used painkillers (AMD: -0.09, CI 95%: -0.55 to 0.36, P = 0.66). Although the episiotomy pain improved during the wound healing process over time, no significant difference in pain perception was observed between the two groups during the intervention (Table 3). No side events were reported in both groups.

**Table 3:** The comparison of mean score of pain in probiotic and placebo groups over time

Group Time	Probiotic group (n = 34) Mean (SD)	Placebo group (n = 35) Mean (SD)	Mean difference (95% Confidence interval)	P-value
**Before discharge**	4.05(2.38)	3.97(1.44)	0.08(-0.03 to 0.20)	0.68*
**5 ± 1 days after birth**	1.47(0.64)	1.95(0.35)	-0.47(-0.97 to 0.19)	0.059*
**15 ± 1 days after birth**	0.37(0.17)	0.46(0.22)	-0.09(-0.55 to 0.36)†	0.66**

†Adjusted mean difference after controlling the effect of used painkillers, *Independent t –test, **RMANOVA

## Discussion

This is the first study, which evaluated the effect of Lactobacillus casei on episiotomy wound healing. The results of the present study indicated that the use of Lactobacillus casei oral supplementation was effective in episiotomy wound healing. Consistent with the findings of the present study, Abootaleb et al. [13], revealed that daily spraying of Lactobacillus casei on second-degree burn wounds reduced inflammation and accelerated wound healing. In another study, the effect of probiotic supplementation on burn wound of 40 children was evaluated. Participants were randomly divided into intervention group (receiving topical combined probiotics containing Lactobacillus fermentum and Lactobacillus delbruekii, twice a day with silver sulfadiazine ointment) and placebo group with silver sulfadiazine topical ointment. The results indicated that wound healing was significantly accelerated in the probiotic receiving group [25].

The findings of a study indicated no difference in the treatment and healing process of grade 2 infectious and grade 3 non-infectious burns using Lactobacillus plantarum or silver sulfadiazine ointment. Additionally, the use of Lactobacillus plantarum was suggested as an alternative treatment for burns, since it had a similar effect to classical treatment of burn wounds. However, due to the small sample size and low statistical power of the study, it was recommonded to conduct further studies with a large sample size [24].

In line with the findings of the present study, Sinha et al. [26] investigated the effect of topical probiotic gel prepared from VITSAMJ1 metabolites, extracted from goat milk, on skin and subcutaneous wound healing in the back of female Wistar rats in India. They revealed that using topical probiotic gel twice a day accelerated the wound healing process compared with the negative control group (receiving glycerine and glycerol) and the control group (without treatment).

In a study in Brazil, the use of combined oral probiotics (L. paracasei, Bifidobacterium lactis HN0019, L. rhamnosus HN001, and L. acidophilus) after surgery at a dose of 250 mg a day for 15 days resulted in faster wound healing in rats compared with placebo group, as reducing the inflammatory phase, accelerating the fibrosis process, and collagen deposition were mentioned as the mechanism of its possible effect [33]. Furthermore, the use of Lactobacillus brevis on skin wound healing in the back of male Wistar rats demonstrated that the percentage of inflammation reduction and wound healing on the fourteenth day was significantly higher in the probiotic group compared with no treatment group [34].

Twetman et al. [35] in a pilot study evaluated the effect of suction lozenges containing Lactobacillus reuteri DSM 17,938 and ATCC PTA 5289 on oral wound healing of ten volunteer students. The findings indicated no significant difference in wound healing during days 5 and 8 after the intervention in the probiotic group compared with the placebo group, which is inconsistent with the results of the present study. It was recommended to conduct further studies with a large sample size.

In the present study, the perceived pain in the intervention group was less than that in the control group on 5 ± 1 and 15 ± 1 days after birth. However, this decrease was not statistically significant. Consistent with the findings of the present study, in the study of Twetman et al. [35] the use of suction lozenges containing Lactobacillus reuteri demonstrated no significant difference in oral wound pain of intervention group compared to that of the placebo group.

Inconsistent with the findings of the present study, Lei et al. [36] in the relief effect comparison of Lactobacillus casei Shirota and placebo on the pain of single rib fracture among 283 eligible individuals reported a significant reduction in intensity of pain perception in the probiotic group compared with that of placebo group. Further, they recommonded to conduct further studies on its possible mechanisms.

In addition, the results of a laboratory study indicated that taking 1 cc of combined supplementation of probiotics (Lactobacillus plantarum, Lactobacillus delbrueckii, Lactobacillus acidophilus, Lactobacillus rhamnosus, and Bifidobacterium bifidum) daily for up to 21 days had a relief effect on induced neuropathic pain of the sciatic nerve of rats, as its possible mechanism has been attributed to the antioxidant properties of probiotics [37]. The contradictions of the findings of the present study with other studies can be attributed to the different types of pain studied, different types of probiotics, differences in pain threshold and pain tolerance in different people, and self-reported data for pain.

### Strengths and limitations

The study design including randomized clinical trial, and allocation concealment were among the strengths of the present study. The results of the present study should be considered in the light of some limitations, including small sample size. Another limitation of the present study as other self-statement ones is the perception of pain severity that is variable among people.

## Conclusion

Lactobacillus casei oral supplementation is effective in episiotomy wound healing with no significant impact on pain relief. It is recommended to evaluate the effect of topical use of Lactobacillus casei on episiotomy repair and pain in further studies.

## Data Availability

Data sets used and/or analyzed during the current study are available from the corresponding author upon reasonable request.
